# Development of an anti-infective coating on the surface of intraosseous implants responsive to enzymes and bacteria

**DOI:** 10.1186/s12951-021-00985-3

**Published:** 2021-08-12

**Authors:** Xin Liao, Xingfang Yu, Haiping Yu, Jiaqi Huang, Bi Zhang, Jie Xiao

**Affiliations:** 1 The Second Affiliated Hospital (Jiande Branch), Zhejiang University School of Medicine, Jiande, Hangzhou, Zhejiang China; 2 Department of Orthopedics, The Affiliated Yiwu Hospital of Wenzhou Medical University, 699 Jiangdong Road, Yiwu, 322000 Zhejiang China

**Keywords:** Intraosseous implants, Infections, Multilayer films, Microenvironment, Antibacterial effect

## Abstract

**Background:**

Bacterial proliferation on the endosseous implants surface presents a new threat to the using of the bone implants. Unfortunately, there is no effective constructed antibacterial coating which is bacterial anti-adhesion substrate-independent or have long-term biofilm inhibition functions.

**Methods:**

Drug release effect was tested in Chymotrypsin (CMS) solution and *S. aureus*. We used bacterial inhibition rate assays and protein leakage experiment to analyze the in vitro antibacterial effect of (Montmorillonite/Poly-l-lysine-Chlorhexidine)_10_ [(MMT/PLL-CHX)_10_] multilayer film. We used the CCK-8 assay to analyze the effect of (MMT/PLL-CHX)_10_ multilayer films on the growth and proliferation of rat osteoblasts. Rat orthopaedic implant-related infections model was constructed to test the antimicrobial activity effect of (MMT/PLL-CHX)_10_ multilayer films in vivo.

**Results:**

In this study, the (MMT/PLL-CHX)_10_ multilayer films structure were progressively degraded and showed well concentration-dependent degradation characteristics following incubation with *Staphylococcus aureus* and CMS solution. Bacterial inhibition rate assays and protein leakage experiment showed high levels of bactericidal activity. While the CCK-8 analysis proved that the (MMT/PLL-CHX)_10_ multilayer films possess perfect biocompatibility. It is somewhat encouraging that in the in vivo antibacterial tests, the K-wires coated with (MMT/PLL-CHX)_10_ multilayer films showed lower infections incidence and inflammation than the unmodified group, and all parameters are close to SHAM group.

**Conclusion:**

(MMT/PLL-CHX)_10_ multilayer films provides a potential therapeutic method for orthopaedic implant-related infections.

## Introduction

Extremity fracture from high energy trauma need reduction and internal fixation (ORIF) to stabilise the injury, traditionally use endosseous implants to treatment fracture [1–3]. However, bacterial proliferation on the iendosseous implants surface and succedent biofilm formation present a new threat to the using of the implants [4, 5]. For example, extensive interventions including multiple rounds of mass antibiotics treatment, infection rate after Gustilo-Anderson Type III fractures can rise to almost 52% [6]. More seriously, only the United States occurs about 17 million cases of biofilm-related infectious diseases every year, directly cost about90 billion US dollars, and the most important one is the implants infection [7]. In the presence of implants, trace bacteria can cause infections, at the same time, common bacteria that cause infections, such as Staphylococcus aureus, have a strong affinity for various orthopedic implants materials, and are easy to adhere to the surface of the materials and cause infection [8, 9]. Compromised local immunity also encourage bacterial proliferation. Afterwards, these bacteria proliferate and develop the biofilm itself [10, 11]. So, a multiple effort has been paid to research antibacterial surfaces either to directly eliminate bacteria or resist bacterial attachment surrounding the endosseous implants for long-term biofilm inhibition [12–14]. Unfortunately, there is no effective constructed antibacterial coating which is bacterial anti-adhesion substrate-independent or have long-term biofilm inhibition functions [15–17]. Once bacteria adhesion transferred to the irreversible stage, bacterial overgrowth and biofilm formation are ineluctable [18].

Academic researchers show that after tissue infection, the microenvironment changes due to pH decreases, hypoxic metabolism and abnormal expression of enzyme [19–21]. So the delivery systems which possess the function of responsive drug release are highly favored. They are sensitive to different environmental changes, and release the drug when needed to work. The stimulus factors of responsive release include pH, enzymes, biological systems, ight, redox, electric, temperature and magnetic [22–25]. Although researchers have done a lot of work in this area, the delivery of antibiotics in a bacterial self-defense and on-demand way is rare [26, 27]. Therefore, the use of the microenvironmental characteristics formed after bacterial infection to prepare smart antibacterial coatings that can achieve microenvironmental response to achieve on-demand and precise release of antibacterial drugs at local infection sites is a hot research topic.

Layer-by-layer self-assembly is a means of forming nano-structured multilayer films through alternate deposition of materials based on the interaction force between materials [28, 29]. As a novel material preparation technology, layer-by-layer self-assembly has many advantages such as controllable preparation conditions, suitable for a variety of substances, and industrialization prospects [30, 31]. It has become a very important method of constructing composite functional films and drug carriers in medical materials. According to the nature of the material, different materials can be selected to achieve different response effects. At the same time, under the stimulus of external conditions, the intelligent multilayer film can realize the selective embedding of substances [32]. The intelligent controlled release of the drug can not only reduce the toxic effect of the drug in the human body, but also maintain the drug concentration within the effective therapeutic concentration range and prolong its in vivo/in vitro action time [33, 34]. According to different release mechanisms, these response means can be divided into enzyme response, pH response, light response, temperature response, etc. [35].

Researchers have made great progress in the preparation of antibacterial coatings on the surface of orthopedic materials using layer-by-layer assembly technology. For example, Wang et al. prepared drug-carrying chitosan microspheres by emulsification, and then filled chitosan microspheres containing vancomycin in specially treated titanium micropores by infiltration and solidification, and passed in vitro antibacterial experiments [36]. It has been verified that it has a certain antibacterial effect on Staphylococcus epidermidis. Lv et al. used covalent grafting to graft a coating containing chitosan on an aminated modified titanium plate, using tetracycline as a model drug, and the results showed that chitosan can effectively increase the drug loading and antibacterial properties of the coating [37]. The titanium metal antibacterial coating system constructed by Kumeria uses both chitosan and polylactic acid. They use electrochemical corrosion technology to corrode titanium nanotubes on the surface of titanium metal as the carrier of gentamicin. The surface of titanium nanotubes was coated with chitosan and polylactic acid by dipping in a lactic acid mixed solution, and it was found that the gentamicin-loaded titanium metal after surface modification by chitosan and polylactic acid can play a better antibacterial effect, and can promote the adhesion of osteoblasts [38].

Staphylococcus aureus is the most common pathogens in endosseous implants infection. And the indiscriminate use of antibiotics has resulted in bacteria developing resistance to antibiotics [39]. And non-controllable drug-loaded antibacterial coatings cannot achieve the prevention of mid- and late-stage infections, and also can cause bacteria to develop resistance to antibiotics. With a rise in antimicrobial resistance, researchers have begun to study alternative bactericidal compounds [40]. Previous studies have shown that chlorhexidine (CHX) has the great antibacterial efficacy, specially against gram-positive bacteria, and is used in different fields of medicine [41]. Studies have shown that when bacteria infect the host, the expression of pathogenic factors such as Chymotrypsin (CMS) and hyaluronidase (HAS) in the infected microenvironment is significantly increased, forming a special bacterial infection microenvironment [42, 43]. When infections occurred in the surrounding environment, the MMT/PLL multilayer film could disintegrate and actively released the loaded drugs to realize intelligent antibacterial [44]. Montmorillonite (MMT) is a negatively charged natural mineral with a thickness of nanometers [45]. It has good adsorptivity, can adsorb bacteria in water, and has low toxicity. It is considered a suitable antibacterial carrier. As a new type of natural bacteriostatic agent, poly-l-lysine (PLL) has broad-spectrum antibacterial activity against most Gram-negative and Gram-positive bacteria, fungi and viruses [46]. We prepared CHX-loaded coatings (MMT/PLL-CHX)_10_ consisting of montmorillonite (MMT), poly-l-lysine (PLL), and CHX via electrostatic interactions. The CMS secreted by bacteria can promote the degradation of the multilayer film structure, actively release antibacterial agents, and achieve precise and efficient sterilization effects.

## Materials and methods

### Reagents and materials

Poly-l-lysine hydrobromide (PLL, Mw: 4000–15,000 by viscosity), polyethyleneimine (PEI, Mw: 25 kDa), chymotrypsin (CMS, α-chymotrypsin from bovine pancreas, Type II, ≥ 40 units/mg protein) and CHX were bought from Sigma-Aldrich. LIVE/DEAD® Viability/Cytotoxicity Kit (Invitrogen, L3224) was purchased from Intergen (Purchase, NY). Titanium Kirschner wires (K-wires, 1.25 mm) was purchased from MK Medical GmbH & Co. Silicon wafer substrates wre purchased from Si-Mat. Phosphate buffered saline (PBS) 10X was obtained from Gibco® Life Technologies. LB agar and LB broth were from Hopebio (China).

### Construction of the (MMT/PLL-CHX)_10_ multilayer films

We fabricated multilayer thin films by conventional LbL self-assembly. The glass discs, silicon wafer substrates, and K-wires were ultrasonically cleaned in acetone and then in ethanol for 2 h, dried in cold air. MMT stock solution (5 mg/mL) was also prepared 15 days in advance. The MMT stock solution was diluted with deionized water to a final concentration of 0.5 mg/mL, which was dispersed using ultrasonic treatment overnight. After that, PLL and CHX in deionized water were dissolved at 1.0 mg/mL and 1.0 mg/mL separately for preparing (MMT/PLL-CHX)_10_ multilayer film deposition. More specifically, Substrates were sterilized firstly in PEI solution (5 mg/mL) at room temperature for 30 min for a precursor. Then dipped the substrates in the MMT solution for 20 min and then rinsed five times with buffer solution. The films were dried in a stream of N2 gas. We next dipped the substrates in PLL-CHX solution for 20 min, followed by rinsing five times with buffer solution. This cycle produced a single bilayer of positively and negatively charged polyelectrolytes. The sample was labelled here as (MMT/PLL-CHX)_1_. We consecutively repeated the deposition process until the (MMT/PLL-CHX)_10_ multilayer films were fabricated.

### Characterization of the multi-lamellar membrane structure

Detailed descriptions about the preparation of (MMT/PLL-CHX)_10_ multilayers have been described. First, the thickness and frequency of material deposited in each layer was obtained by a QCM (QCM200, 5 MHz, Stanford Research Systems). Apart from this, we used Hitachi S-4800 electron microscope (Tokyo, Japan) to examine the morphology of the multilayer films structure at an accelerating voltage of 10 kV. A sample of each multilayer films was previously sputtered with a gold–palladium mixture for five minutes under vacuum. Finally, adjusted the image so that the multilayer films structure was clearly visible and took a photographic record. Finally, Zeta potential measurements were performed by a zeta potential analyzer (ZS90, Malvern Instruments Ltd., Malvern, UK).

### Drug release from the (MMT/PLL-CHX)_10_ multilayer films

In order to evaluate the CHX release from the (MMT/PLL-CHX)_10_ multilayer films structure. 3 cm (MMT/PLL-CHX)_10_ multilayer films-coated K-wires were decanted separately in 30 mL phosphate buffered saline (PBS), different concentration of CMS solution and different concentration of *S. aureus* at at 37 °C. At specific time intervals, we used spectroscopy (Synergy 2, BioTek, Winooski, VT, USA) to test CHX concentration at 231 nm.

### Responsive degradation of the (MMT/PLL-CHX)_10_ multilayer films

In this experiment, silicon wafers were selected as substrate material. The fabricated(MMT/PLL-CHX)_10_ multilayer films were deposited in 0.01 M PBS, CMS solutions, and *Staphylococcus aureus* (*S. aureus*, ATCC 27217) for 3 days. All samples were taken at the same time and dried under nitrogen atmosphere. Then the samples were sterilized under UV light at 254 nm for 30 min placed 25 cm from the samples. We displayed the responsive degradation of the (MMT/PLL-CHX)_10_ multilayer films through zone of bacterial inhibition (ZOI) and the sample thickness. Specifically, Luria–Bertani (LB) broth and LB agar (1.5% agar) were used to culture *Staphylococcus aureus.* 100 µL of freshly grown overnight Staphylococcus aureus were spread over sterile nutrient agar plates, evenly coated. The prepared samples were placed onto agar plates, and incubated overnight in a 5% carbon dioxide incubator at 37 °C. After incubation, the ZOI was measured with a ruler and recorded in centimeter and taken as the marker for multilayer films antibacterial activity. Meanwhile, the thickness of all samples after incubation were followed by spectroscopic ellipsometry (M-2000 DITM, J.A. Woollam). According to published standard methods [47], the continuing wavelength ranging from 124 to 1700 nm and selected the angle of incidence of both 65ºand 70º for ellipsometry measurements. We choosed ∆ and Ψ values surveyed at a wavelength of 600–1700 nm for analysis. The thickness of samples was determined through the Cauchy model. We set parameters An and Bn for the Cauchy layer at 1.45 and 0.01, respectively, as fit parameters. Then, the thickness that fit the multilayer films was fabricated such that it can be automatically calculated.

### In vitro antimicrobial assays

#### Bacterial inhibition rate assays

The shake-flask culture method with *S. aureus* was used to to test the antibacterial effect of the (MMT/PLL-CHX)_10_ multilayer films according to the previous reports. In this experiment, Polydimethylsiloxane (PDMS) was selected as substrate material, the (MMT/PLL-CHX)_10_ multilayer films was placed on the PDMS and dried at room temperature. First, all the samples were sterilized under UV light at 254 nm for 30 min. Then we placed the (MMT/PLL-CHX)_10_ multilayer films coated with PDMS into sterilized test tubes, containing with 10 mL of 2.5 × 10^4^ CFU/mL of S. aureus solution suspension in saline solution (vehicle, 0.9% NaCl).The unmodified PDMS was set as control group. Afterward, these test tubes were incubated in a shaker incubator for 24 h at 37 °C. S. aureus were pipetted from the test tubes described above and used to prepare consecutive dilutions by taking 0.1 mL of the original solution, then mixed with 9.9 mL of PBS. Next, 100 L *S. aureus* solution from the above solution was plated on solid agar and repeated five times for each group. Following incubation for 24 h, the viable number of *S. aureus* colonies was counted and reported as mean (CFU/mL).

#### Protein leakage experiment

In vitro grown *S. aureus* were spun down for 10 min at 6000 rpm at 4 °C, rinsed three times with normal saline, and diluted in normal saline to get OD600 values of 1.5. One cubic centimeter of (MMT/PLL-CHX)_10_ multilayer films PDMS was put in 20 mL of bacterial fluid. The control group add unmodified PDMS sample. The above mixture was put in a shaking incubator (500 rpm) at 37 °C for 4 h. Put forty microliters (40 µL) of supernatant into 400 µL of working solution, and protein concentration was measured by the Pierce BCA Protein Assay Kit. And the incubation was continued for an additional 30 min, the absorption of the above mixed solution was determined at 562 nm wavelength in order to calculate the protein leakage concentrations.

#### Resistant bacteria experiment

The improper use of antibiotics promotes the development of antibiotic-resistant bacteria. And the emergence of resistant bacteria has developed into a world-wide public health concern. In this regard, researchers have done a lot of work [48]. In this study, we selected the MRSA (ATCC43300). In particular, 24-well plate was selected as substrate material, (MMT/PLL-CHX)_10_ multilayer films were prepared. The antibacterial activity of the membranes against MRSA was evaluated by inhibition zone.

### Cell counting kit-8 assay

In this study, we used the CCK-8 assay according to the manufacturer’s instructions to analyze the effect of (MMT/PLL-CHX)_10_ multilayer films on the growth and proliferation of rat osteoblasts. Primary rat osteoblasts were isolated through a method described previously [49]. In order to test the biocompatibility of the (MMT/PLL-CHX)_10_ multilayer films, the extracts of multilayer films were obtained according to the guidelines specified in ISO10993-12:2012 [50]. Used the 64 g/L phenol solution as a control. Briefly, the osteoblasts were grown in DMEM medium supplemented with 100U/mL penicillin, 10% fetal bovine serum and 100 µg/mL streptomycin in a standard incubator. Confluent cells were digested by 0.25% trypsin-0.02% EDTA, then followed by centrifugation (1000*g* for 3 min) in order to harvest the cells. About 1.0 × 10^4^ osteoblasts cells were seeded by using 96-well cell culture plates. Following a 24-h incubation, the culture medium was removed and phenol solution (200 µL/well) and extracts were added. At 4 and 7 days, the CCK-8 reagent was added into each plate well and incubated for 2 h. The absorbance was determined at a wavelength of 450 nm. Six repeats were performed at each time point.

### In vivo antibacterial efficacy study

#### Animals and animal preparation

Thirty male Sprague–Dawley rats (age of 4 months, body weight of 280 ± 20 g) were obtained from the Animal Administration Center of Wenzhou Medical University. Animal care, operation, treatment procedures, and animal welfare were executed in strict accordance with the National Institutions of Health Guide for the Care, with relevant study programs also approved by the Animal Care and Use Committee of Wenzhou Medical University. After the rats had been adaptively fed for 14 days in room temperature adaptability conditions, the experiment was conducted. More specifically, rats were randomly divided into three different groups (10 animals per group). Firstly, we prepared unmodified Kirschner wires and (MMT/PLL-CHX)_10_ multilayer film coated Kirschner wires. All the Kirschner wires were sterilized under UV light at 254 nm for 30 min, stored in a sterile, sealed storage box. All rats were intraperitoneally anesthetized with chloral hydrate 10% anesthesia (chloral hydrate 10%/rat body weight = 0.35 mL/100 g). A broad area of the left knee joints was shaved, and the underlying skin washed with a povidone-iodine solution, wiped with 70% alcohol, and draped for surgery. Then, the skin, subcutaneous tissue and joint capsule was cut, exposing the tibial plateau. It is worth noting that important nerves, muscle, blood vessels, ligamentous tissues and anatomical structural should be protected as much as possible. After the tibial plateau was fully exposed, under strictly sterile conditions, the medullary cavity was drilled vertically with a prepared 0.8 mm K-bit, drilled perpendicular to the tibial plateau of the rats and small volumes of bone marrow was aspirated using a sterile syringe. The K-wire was placed in the borehole as quick and as accurate as possible. The first group was placed with unmodified Kirschner wires, and 10 µL of the 10^6^ cells/mL S. aureus suspension was inoculated in the above borehole (unmodified group). The second group was placed with (MMT/PLL-CHX)_10_Kirschner wires, and 10 µL of the 10^6^ cells/mL *S. aureus* suspension was inoculated in the above borehole ((MMT/PLL-CHX)_10_ group). The third group was placed with unmodified Kirschner wires, and no injection of *S. aureus* suspension (SHAM group). All boreholes were subsequently closed with bone wax and the wound were stitched with 3–0 interrupted nylon sutures. Strictly followed the requirements of surgical aseptic operation throughout this operation. The successfully resuscitated rats were monitored for 1 h. All rats were raised under standard breeding conditions and monitored daily.

#### Inflammation indicators

Blood was examined for total WBC counts, CRP levels, IL-1 and IL-8, they are strong important inflammatory indicators.

#### X-ray and bacteriological examination

Used the small-animal X-ray fluorescence tomography (energy 45 kV, current 250 mA, integration time 200 ms, Carestream DRX) to inspect and evaluate the metaphysis of tibial plateau. After the rats were killed, the knees wound were examined carefully, and then collected respective tissue fluid for the bacteriological examination. The Kirschner wires were removed from the borehole, washed and stained according to the kit protocol. In addition, Kirschner wires were sonicated in prepared sterile PBS solution for 40 min. The supernatant from the K-wires were serially diluted in sterile saline, plated onto agar plate media (Thermo Fisher Scientific), and incubated at 37 °C for 24 h. Lastly, *S. aureus* colonies were counted and normalized to K-wires mass.

#### CT examination

The specimens are fixed in paraformaldehyde for 12 h and then subjected to CT examination. The experimental equipment is a micro-CT system (energy 70 kVp, 114 μA, integration time 300 ms, threshold 220, Skyscan 1173; Skyscan, Kontich, Belgium). We use a ring with a surface radius of 0.1 mm from the metal implant as the volume of interest (VOI). The bone mineral density (BMD), trabecular bone number (Tb.N), percent bone volume (BV/TV), trabecular separation (Tb.Sp), trabecular thickness (Tb.Th) and connectivity density (Conn.D) within the VOI zone are analyzed, 3D and histograms are made according to the built-in software.

#### The tibia specimens bending test

The tibia specimens were wrapped in saline-soaked gauze and stored at − 20 °C freezer (After CT examination). The tibia specimens were removed from the freezer before the test and thawed at room temperature. The tibia specimens were subjected to three-point bending by an ElectroForce 3200 computer-controlled testing machine (Bose Corp., Eden Prairie, USA) to test the integration strength. We chose a crosshead speed of 1 mm min^−1^ for the bending tests until the specimens were broken. Maximum load, Resilience and Resilience stiffness were obtained.

#### Bone tissue HE staining and Masson trichrome

After the tibia specimens bending test, the tibia bones were decalcified with EDTA decalcification solution for one month. Subsequently, the tibia bones were embedded in paraffin. Then part of slices were used for hematoxylin and eosin (H&E) staining. The other portion were then stained with Masson trichrome.

### Statistical analysis

All data in experiment are conducted as the means ± SD. Significant differences between groups were determined through unpaired Student's t-test, post analysis by Tukey’s honestly significant difference test and ANOVA(SPSS 18.0 software, Chicago, IL). P < 0.05 was deemed to indicate statistical significance, P > 0.05 was considered no significant.

## Results and discussion

### Characterization of the multi-lamellar membrane structure

**Fig. 1:**
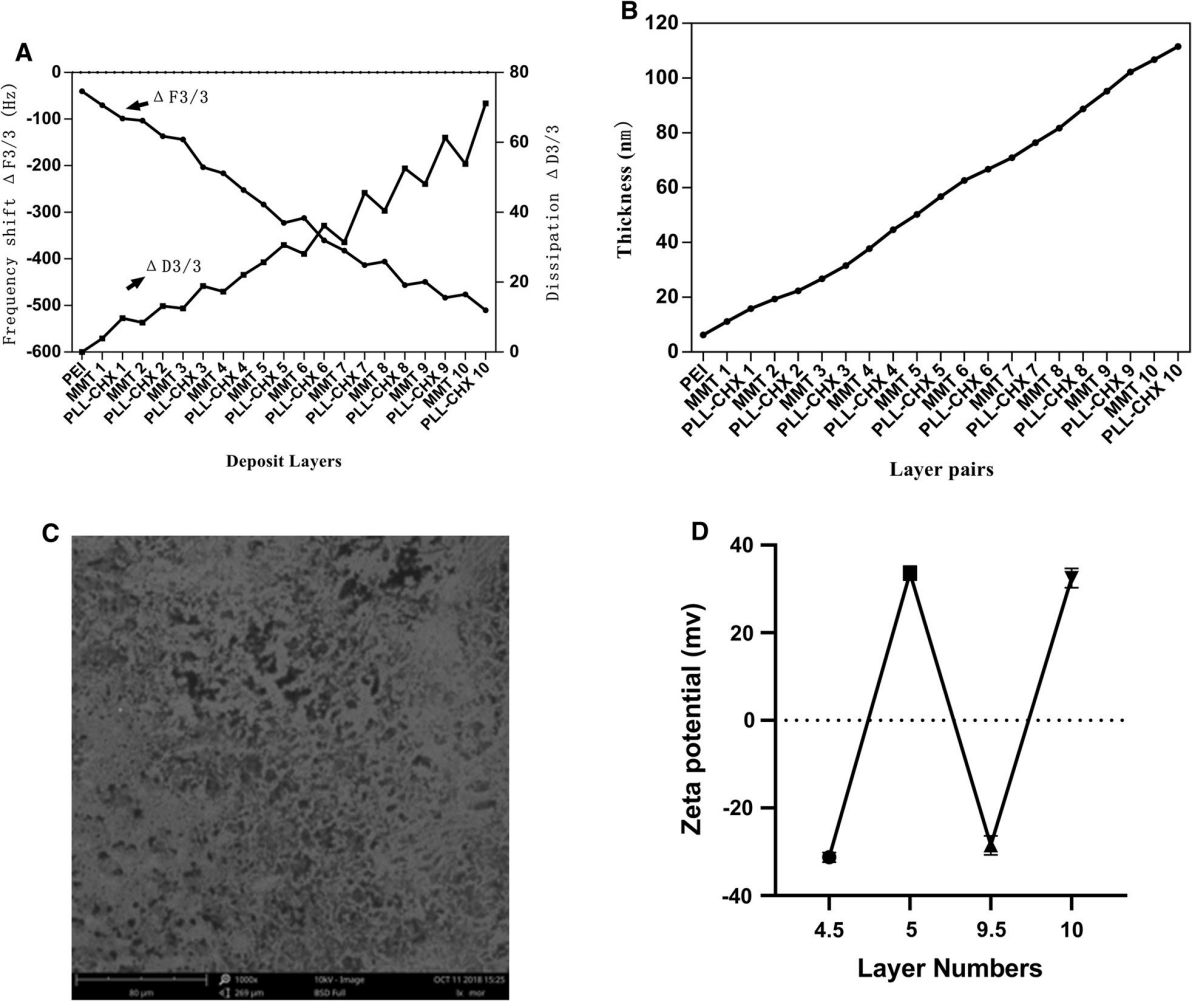
**A** QCM-D data of MMT/PLL-CHX film build-up, frequency shifts and dissipations of overtone n = 3 were presented. **B** Film thickness versis layer pairs calculated by Qsoft. **C** The variation of Zeta-potentials of (MMT/PLL-CHX)_10_ during LBL coating process. **D** SEM images of (MMT/PLL-CHX)_10_ silicon wafer

QCM-D was employed to monitor the LbL assembly of (MMT/PLL-CHX)_10_ multilayers onto flat substrates, and the frequency shifts (∆F) and dissipation differences (∆D) produced at the harmonic of n = 3 were plotted against layers deposited (Fig. 1A). Overall, the frequency shifts decreased and dissipation increased steadily as the deposition step increased. Film thickness was calculated using Q-tools, as illustrated in Fig. 1B, and film thickness increased exponentially as the layer pair number increased. Moreover, Scanning electron microscopy (SEM) was used to analyse the surface morphology of the (MMT/PLL-CHX)_10_ samples. The SEM image revealed that the surface of the (MMT/PLL-CHX)_10_ silicon wafer showed a uniform and porous structure (Fig. 1C). In this situation, we can straightforwardly show that (MMT/PLL-CHX)_10_ multi-layer film structure can be assembled on the surface of silicon wafer.

The zeta potential of (MMT/PLL-CHX)_10_ multi-layer film structure was measured in PBS (Fig. 1D). Half of the layers were deposited layers of MMT, and the whole number of layers were deposited layers of PLL-CHX. Each assembled layer had an alternating positive and negative potential. The real-time monitoring results of zeta potential showed that the (MMT/PLL-CHX)_10_ coating can be successfully assembled.

### Drug release from the (MMT/PLL-CHX)_10_ multilayer films

**Fig. 2:**
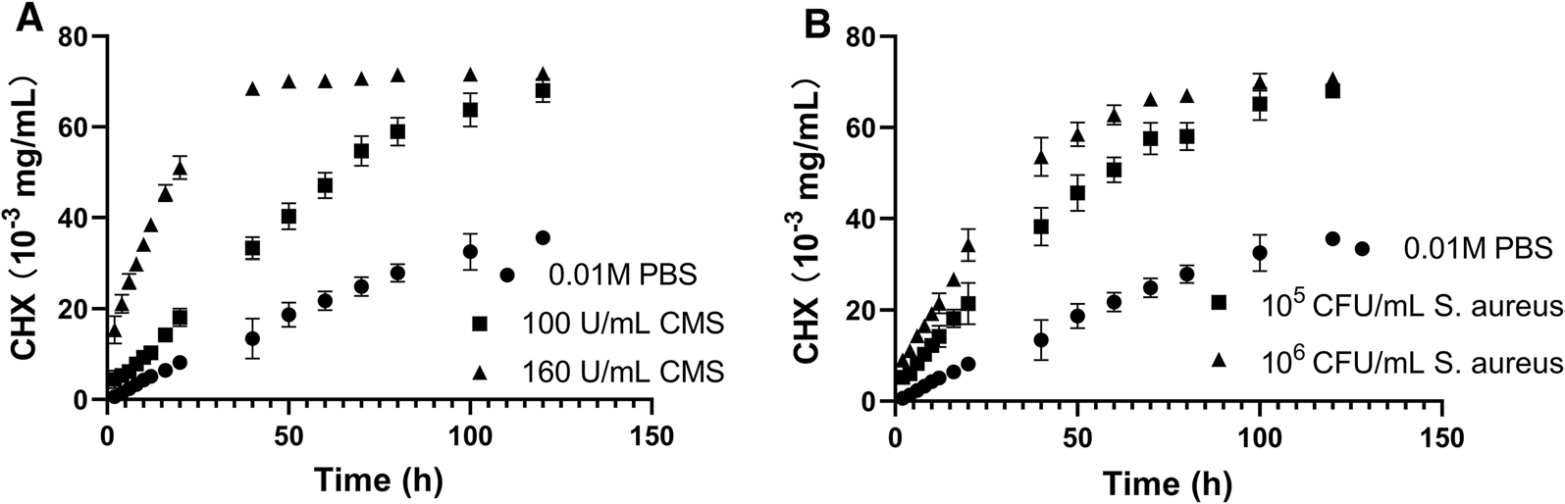
CHX release in **A** different concentration of CMS solution and **B** different concentration of *S. aureus*

The release profile in vitro exhibited a slow CHX release in PBS. Indicated the strong retention property of MMT against CHX release. With the presence of 100 U/mL CMS, the release of CHX from the multi-layer film structure was promoted exhibiting the enzymatic release of the drug. Farther posteriorly, when the concentration of CMS was increased to 160 U/mL, and the released CHX concentration was 51.03 × 10^−3^ mg/mL at 20 h, and by 120 h, the released CHX concentration was 71.77 × 10^−3^ mg/mL (Fig. 2A). Interestingly, analogous phenomena occurred in *S. aureus* (Fig. 2B). The amount of drug released increased with the increased of the concentration of *Staphylococcus aureus*. This might be because bacterial microenvironment could fostered the CHX release based on the CMS secretion and enzymatic degradation of the (MMT/PLL-CHX)_10_ multilayer films.

### Responsive degradation of the (MMT/PLL-CHX)_10_ multilayer films

**Fig. 3:**
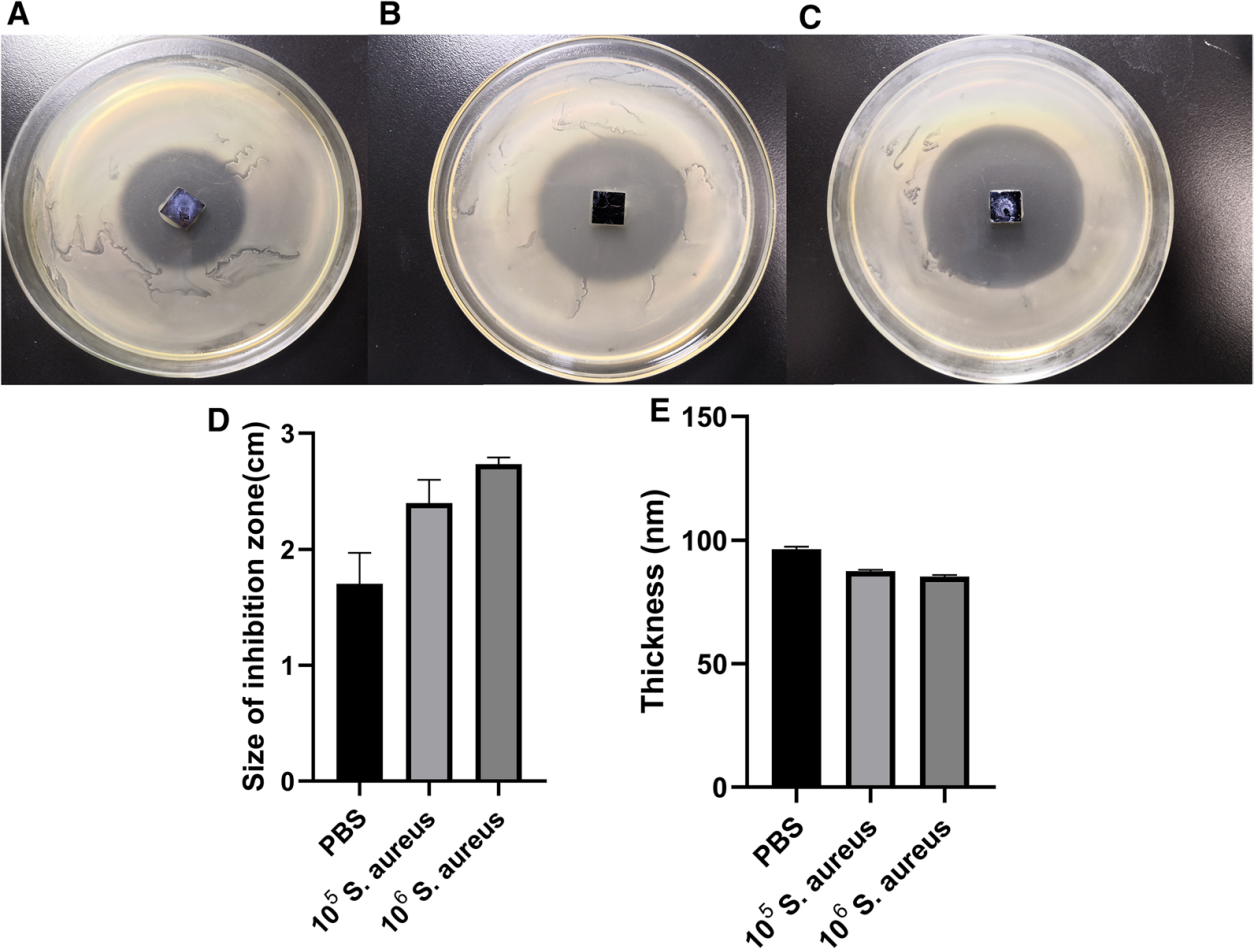
ZOI measurement of (MMT/PLL-CHX)_10_ multilayer films after deposited in **A**–**C** 0.01 M PBS, 10^5^ CFU/mL of *S. aureus* and 10^6^ CFU/mL of *S. aureus*. **D**–**E** Changes of ZOI and thickness

**Fig. 4:**
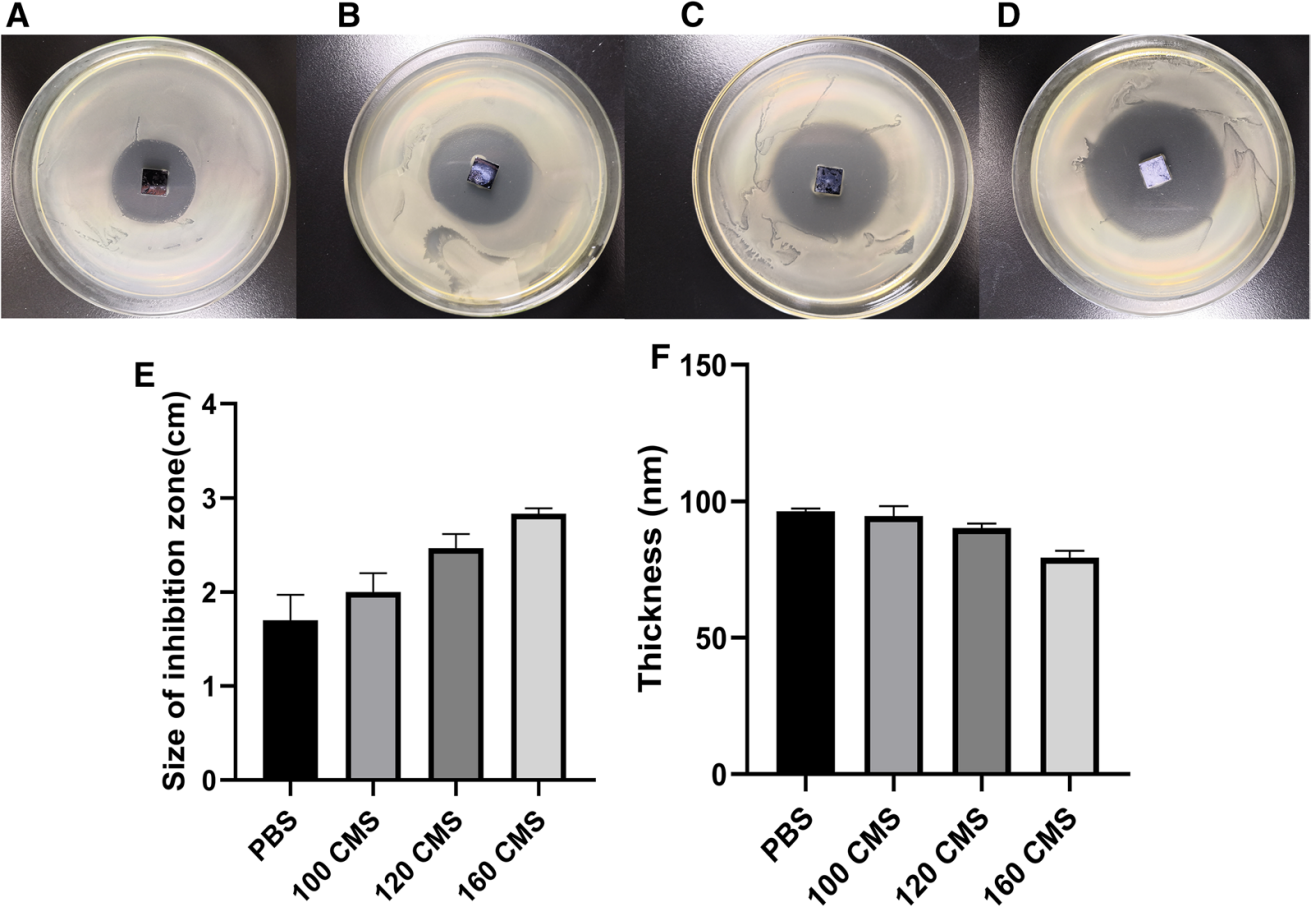
ZOI measurement of (MMT/PLL-CHX)_10_ multilayer films after deposited in **A**–**D** 0.01 M PBS, 100 U/mL of CMS solution, 120 U/mL of CMS solution and 160 U/mL of CMS solution. **E**–**F** Changes of ZOI and thickness

In this experiment, we designed the (MMT/PLL-CHX)_10_ multilayer films which can release CHX when external bacteria multiplied releasing CMS. We used the zone of bacterial inhibition (ZOI) to observe the effectively release CHX depending on the changes in the microenvironment. The results were depicted in Figs. 3 and 4. In more detail, after immersing for 3 days, the ZOI increased with the increase in concentrations of *S. aureus* (Fig. 3A–C). And the statistical graph was presented (Fig. 3D). The 0.01 M PBS solution corresponded to 1.70 ± 0.22 cm, 10^5^ CFU/mL of *S. aureus* solution to 2.40 ± 0.16 cm, and 10^6^ to 2.73 ± 0.05 cm. We also assessed the changes in thickness through spectroscopic ellipsometry (Fig. 3E). We found that the thickness of the (MMT/PLL-CHX)_10_ multilayer films changed from 96.47 ± 0.78 nm to 85.33 ± 0.54 nm. The similar phenomena could also be found when we varied the concentration of CMS. The ZOI increased with the increase in concentrations of CMS (Fig. 4A–D). The 0.01 M PBS corresponded to 1.70 ± 0.22 cm, 100U/mL of CMS solution to 2.00 ± 0.16 cm, 120 to 2.47 ± 0.12 cm, and 160 to 2.83 ± 0.05 cm (Fig. 4E). The thickness also reduced with the increase in concentrations of CMS. The 0.01 M PBS solution corresponded to 96.47 ± 0.78 nm, 100 U/mL of CMS solution to 94.67 ± 2.95 nm, 120 to 90.27 ± 1.40 nm, and 160 to 79.43 ± 2.08 nm (Fig. 4F). The above measurement of ZOI corresponded with the changes of thickness. These phenomena may be attributed to the fact that after the CMS secreted from the bacteria promoted the degradation of the (MMT/PLL-CHX)_10_ multilayer films, most of the multilayer films fragments remained on the surface of the silicon wafer, however due to the degradation of the multilayer films, the CHX was easier to release, therefore, larger ZOI was formed.

### In vitro antimicrobial assays

**Fig. 5:**
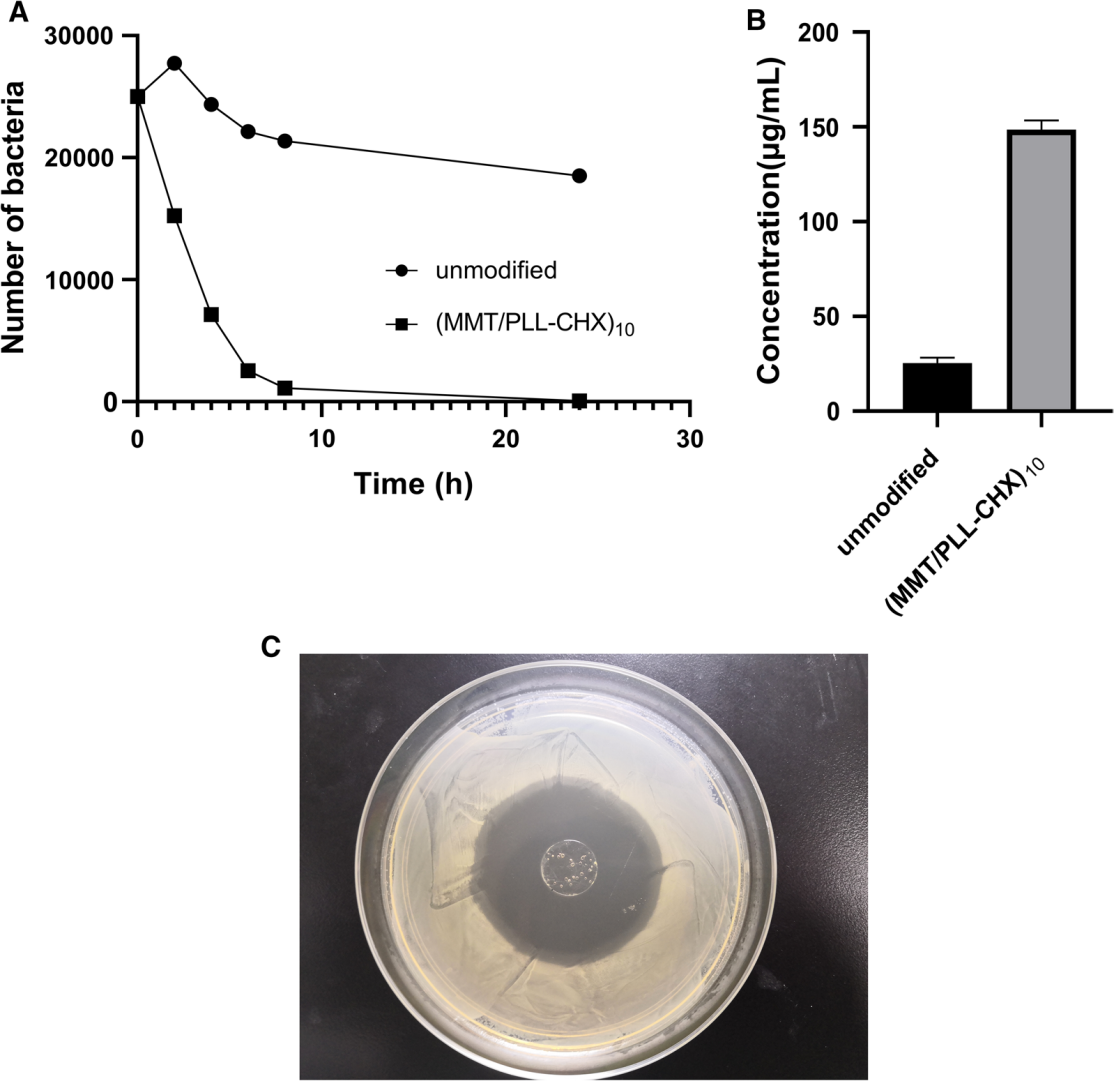
In vitro antimicrobial assays. **A** Bacterial inhibition rate assays. **B** protein leakage experiment. **C** ZOI detection of the (MMT/PLL-CHX)_10_ multilayer films against MRSA

We used bacterial inhibition rate assays and protein leakage experiment to analyze the in vitro antibacterial effect of (MMT/PLL-CHX)_10_ multilayer film. As defined in Fig. 5A, In control group, unmodified PDMS and *Staphylococcus aureus* were co-cultured. It is worth noting that the number of *Staphylococcus aureus* increased significantly in the first 2 h. And slowly decreased during the following 24 h at 37 °C. Finally, We could still find a lot of live bacteria in the test tubes after 24 h. Yet, in (MMT/PLL-CHX)_10_ group, *Staphylococcus aureus* quickly decreased in the first 8 h and then died at 24 h. Apart from this, we also developed protein leakage experiment. The presence of protein in the bacterial suspension indicates damage to the bacterial cell membranes. This experiment we used a BCA Protein Assay Kit to evaluate the amount of protein leakage. The BCA protein assay can form a water-soluble and purple-colored BCA/copper complex with an absorbance at 562 nm. As shown in Fig. 5B, the detected protein concentration with unmodified PDMS was 25.37 ± 2.38 µg/mL for *S. aureus*. However, a higher protein concentration (148.60 ± 3.88 µg/mL) of the bacterial suspension added with (MMT/PLL-CHX)_10_ multilayer films PDMS was found, which was more than 5.86 times than that of unmodified PDMS. This suggested that more leakage of *S. aureus* content had happened. This was due to the functioning of the (MMT/PLL-CHX)_10_ multilayer films PDMS which can enhance bacterial membrane damage capability. The above mentioned experiments might be due to CMS released quickly when outside *S. aureus* strains continued to multiply, which resulted in the rapid degradation of the (MMT/PLL-CHX)_10_ multilayer films, thereby releasing CHX from the system killing *S. aureus* strains rapidly. After calculation, the inhibitory rate of (MMT/PLL-CHX)_10_ multi-layer film was 99%. According to previous studies, CHX has strong bactericidal effects [51]. After incubation, produced obvious zone of inhibition (Fig. 5C). Our study demonstrated the (MMT/PLL-CHX)_10_ multi-layer film structure have good antibacterial property against MRSA.

### Cell counting kit-8 assay

**Fig. 6:**
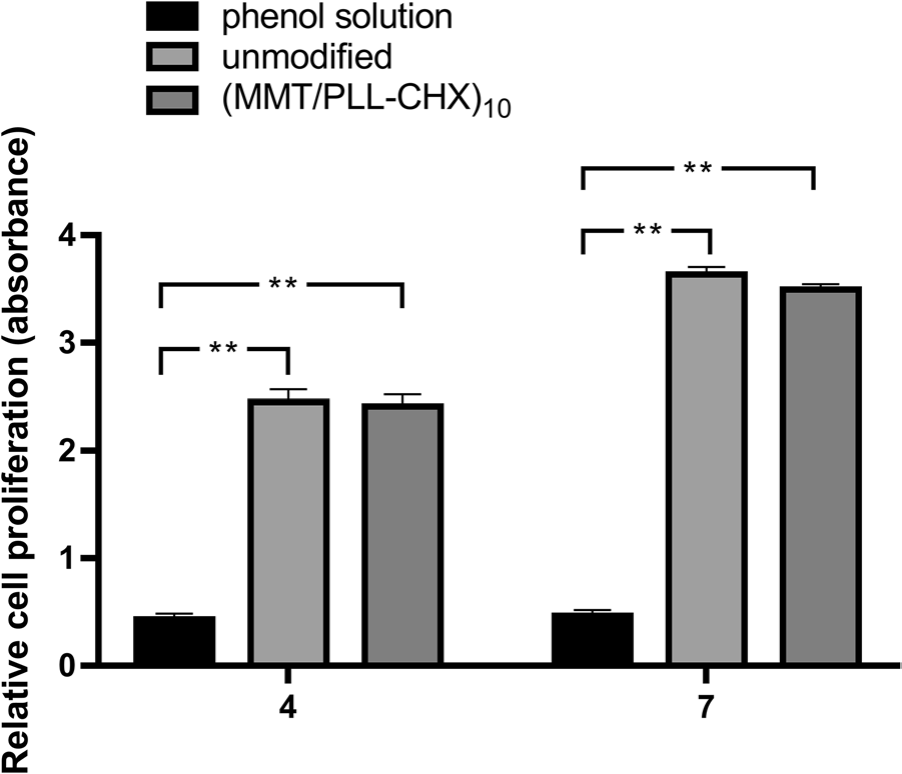
CCK-8 assay for cellular viability

Further, We analyzed whether the (MMT/PLL-CHX)_10_ multi-layer film structure would release cytotoxic substances that affect rat osteoblasts cells survival and proliferation. We used the Cell Counting Kit-8 (CCK-8) assay to assess the effect of above extracts on proliferation of osteoblasts cells. Our experiments showed that cell proliferation was in a time-dependent manner. The (MMT/PLL-CHX)_10_ multi-layer film structure have no toxic effect on normal rat osteoblasts cells and have excellent biocompatibility (Fig. 6).

### In vivo antibacterial efficacy study

#### Inflammation indicators

**Fig. 7:**
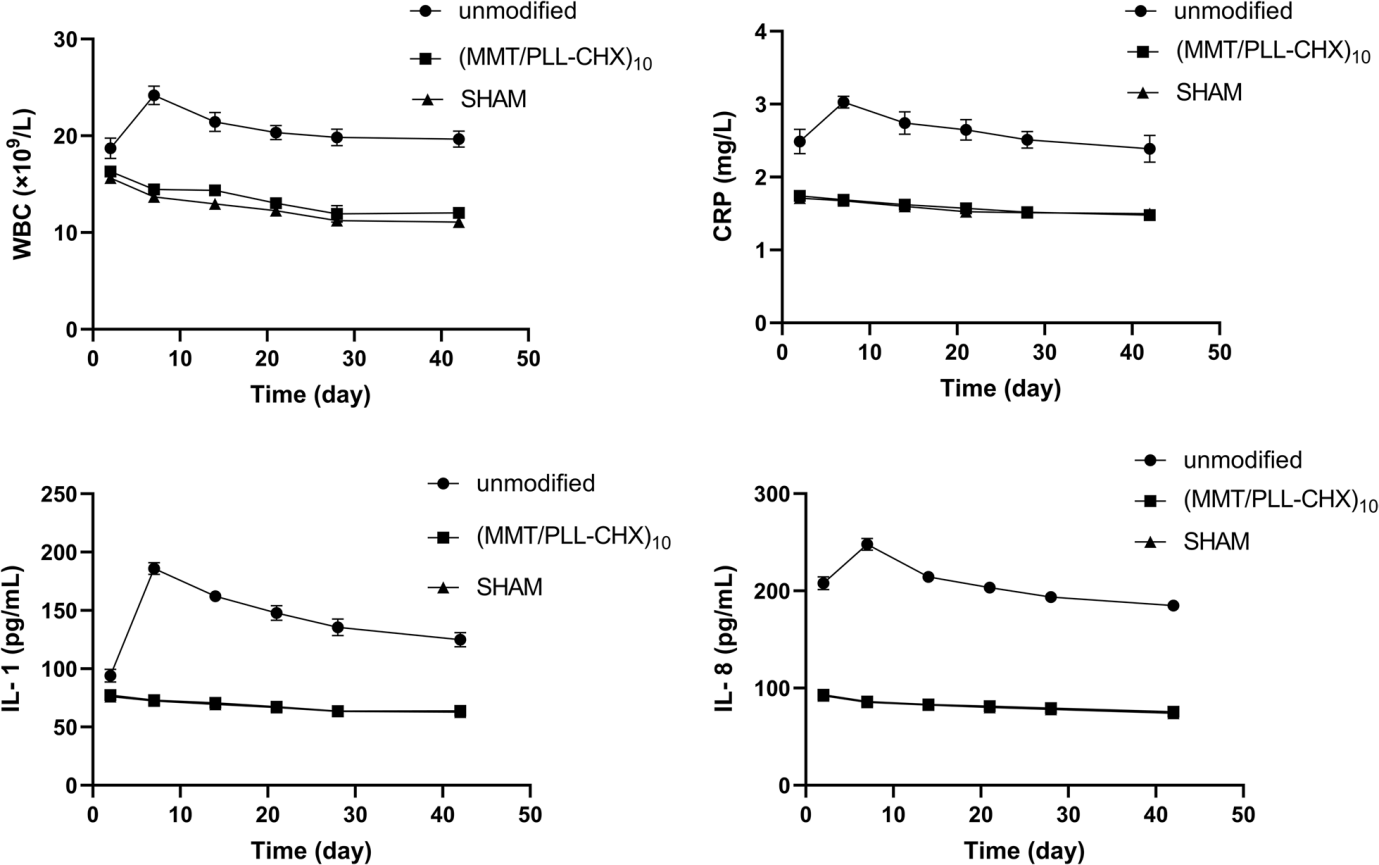
Changes in WBC count, CRP, IL-1 and IL-8 levels of rats with different group

From the 1 day after modeling, all rats returned to their normal condition (Fig. 7). The WBC, CRP, IL-1 and IL-8 for three groups were analyzed. They played an important role in the development of infections. Above infection indicators proved a distinguishing difference between the 3 groups. All infection indicators were increased in 3 groups on the next day after surgery, this may be due to the stress reaction as a result of the surgery. Unmodified group exhibited highest WBC, CRP, IL-1 and IL-8 levels due to lack of CHX and foreign body reactions after 7 days of implantation. Ultimately, all infection indicators of rats in the unmodified group remained higher than normal level after 6 weeks of implantation. This showed that the infection cannot be effectively controlled. Since no bacteria were injected in rats and performed aseptic operation during surgery, no infection occurred in the SHAM group, all inflammation indicators were at normal levels. Interestingly, all inflammation indicators levels in the (MMT/PLL-CHX)_10_ group declined over time and then returned to a normal level after 6 weeks. This certified that (MMT/PLL-CHX)_10_ multilayer film structure had a good anti-infection effect in vivo.

#### X-ray examination

**Fig. 8:**
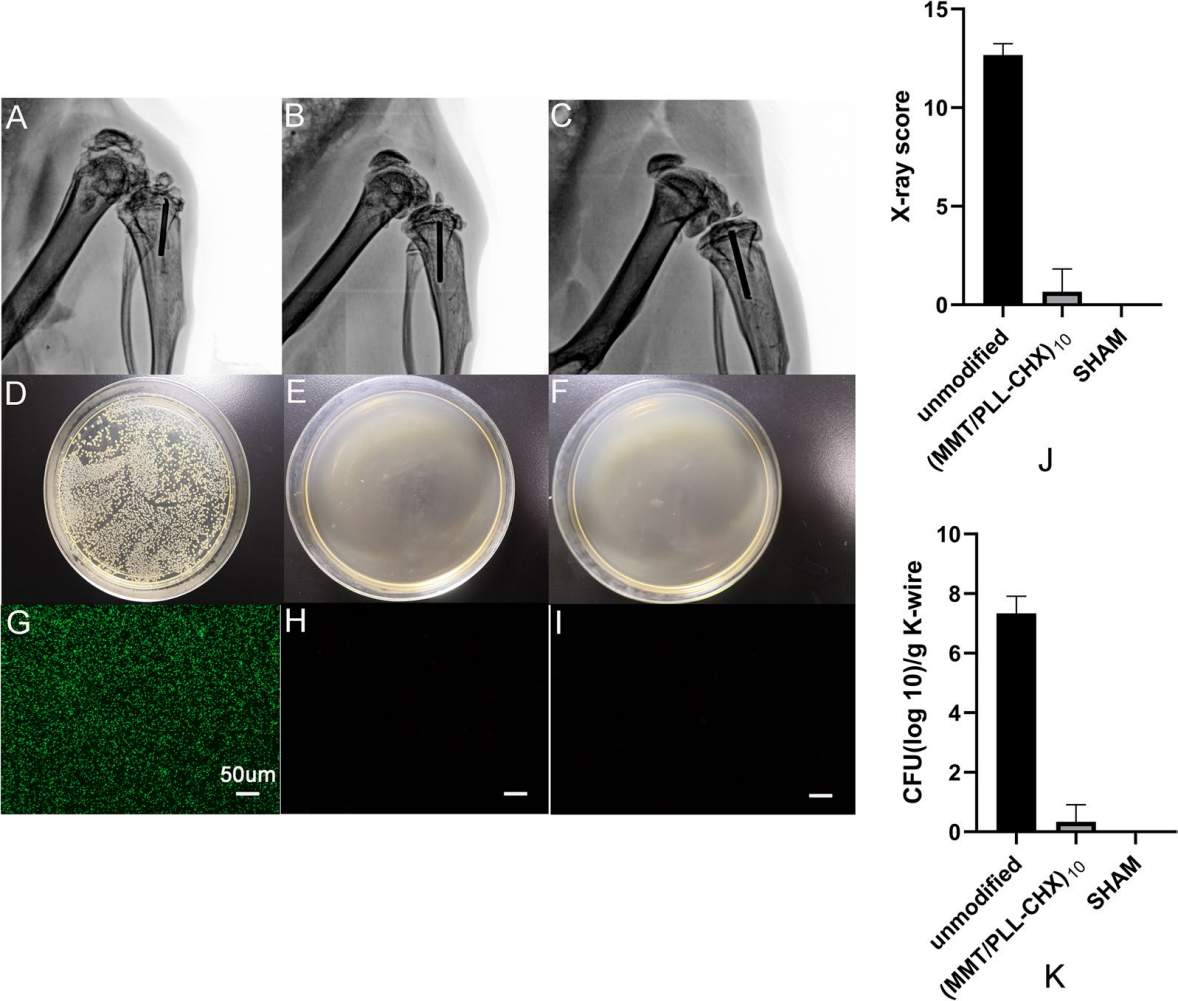
X-ray results after 6 weeks of modeling, **A**–**C** unmodified group, (MMT/PLL-CHX)_10_ group, SHAM group. **J** X-ray score with different group. Bacteriological examination in Kirschner wires, **D**–**F** unmodified group, (MMT/PLL-CHX)_10_ group, SHAM group. **K** bacteria recovered from implanted K-wire. Fluorescent microscopy images of live staining of *S. aureus* adhesions on **G**–**I** unmodified group, (MMT/PLL-CHX)_10_ group, SHAM group

We used the small-animal X-ray fluorescence tomography to inspect and evaluate the metaphysis of tibial plateau in all rats. Because the infection could not be effectively controlled, there observed severe infection in the rat's knee joint of unmodified group. Specifically, the tibial plateau of the unmodified group was characterized by an irregular partially osteolytic lesion, more serious was that adjacent bone tissue is also infected and soft tissue becomes swollen, part of patella, femoral condyle and tibial plateau were translucent (Fig. 8A). This might be caused by the spread of bacteria. However, the (MMT/PLL-CHX)_10_ group and SHAM group showed normal bone and soft tissue morphology with no infection (Fig. 8B, C). To quantify the extent of bone infection, we follow the bone infection radiological evaluation system proposed by Lucke et al. [52]. From the Fig. 8J, We could find that the score of the unmodified group was 12.67 ± 0.47, however the (MMT/PLL-CHX)_10_ group was 0.67 ± 0.94, and the SHAM group was 0. The higher the score, the more serious the infection.

#### Bacteriological examination

In order to further explore the effects of films enzymatic degradation on antibacterial effect in vivo. We performed a detailed bacteriological examination of the samples. After 24 h of culture, we found a lot of bacterias in the tissue fluid of the unmodified group (Fig. 8D). Interestingly, there was no bacterias in the (MMT/PLL-CHX)_10_ group and SHAM group (Fig. 8E, F). More precisely, compared with the unmodified group, there was an average 7 reductions of bacterias in the Kirschner wires of (MMT/PLL-CHX)_10_ group (Fig. 8K).To go a step further, Experimental bacterial viability was determined using the LIVE/DEAD BacLight Bacterial Viability Kit (L-7012, Invitrogen). SYTO9 can stain live bacteria with intact cell membranes to form green fluorescence. As shown in Fig. 8G, there were numerous distinguishable living *S. aureus* cells individually distributed on unmodified Kirschner wires. But, on (MMT/PLL-CHX)_10_ Kirschner wires surface, we could not find any living *S. aureus* cells (Fig. 8H). Since no bacteria were injected, there were still no bacteria here in SHAM group (Fig. 8I).

#### CT examination

**Fig. 9:**
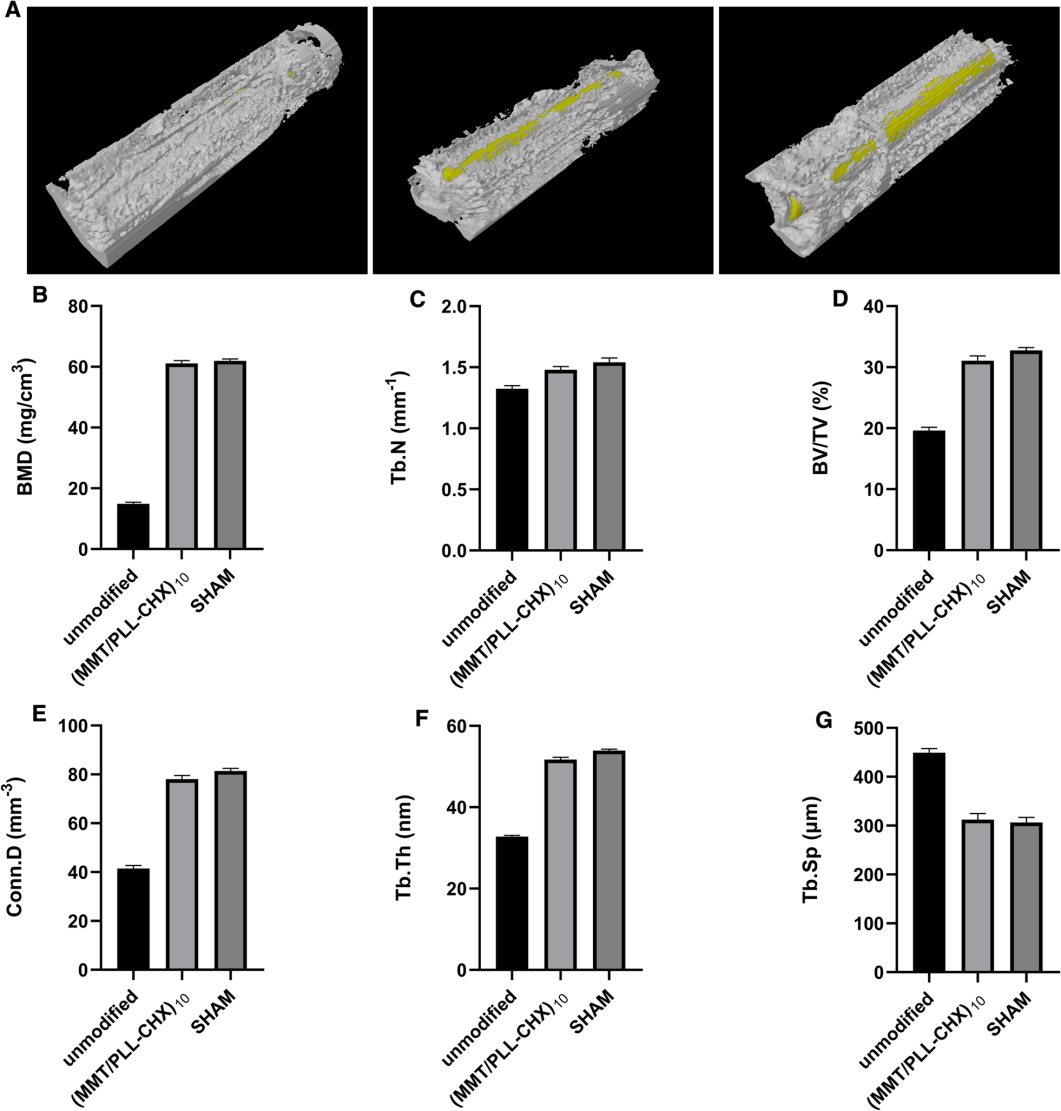
**A** 6 weeks after modeling, micro-CT 3D images of the bone specimens. New bone formation around the Kirschner wires. **B** Bone mineral density (BMD). **C** Trabecular bone number (Tb.N). **D** Percent bone volume (BV/TV). **E** Connectivity density (Conn.D). **F** Trabecular thickness (Tb.Th). **G** Trabecular separation (Tb.Sp)

Intraosseous implant infection can affect the composition of bone tissue. In order to get a more accurate conclusion of the changes of bone composition, we used a micro CT on bone specimens obtained 6 weeks after implantation. Obtained 3D images according to the built-in software, we could obviously observe that both the (MMT/PLL-CHX)_10_ and SHAM group surface promoted new bone formation around the Kirschner wires and enhance osseointegration. Conversely, unmodified group with no new bone formation in Kirschner wires surface (Fig. 9A). Moreover, quantitative evaluation of the trabecular bone within the region of interest (ROI) was showed. Compared with the unmodified implant group, the bone mineral density (BMD), trabecular bone number (Tb.N), percent bone volume (BV/TV), trabecular thickness (Tb.Th) and connectivity density (Conn.D) were significantly higher in the (MMT/PLL-CHX)_10_ implant group (Fig. 9B–F). Conversely, trabecular separation (Tb.Sp) exhibited a significantly lower value in the (MMT/PLL-CHX)_10_ group compared with the unmodified group (Fig. 9G). A nice surprise, all the parameters of the (MMT/PLL-CHX)_10_ group were very close to the SHAM group (P > 0.05). These phenomena could be explained by the “race to the surface theory” in which bone regeneration required a high level of sterility while bacteria would seriously obstruct the bone regeneration process [53].

#### The tibia specimens bending test

**Fig. 10:**
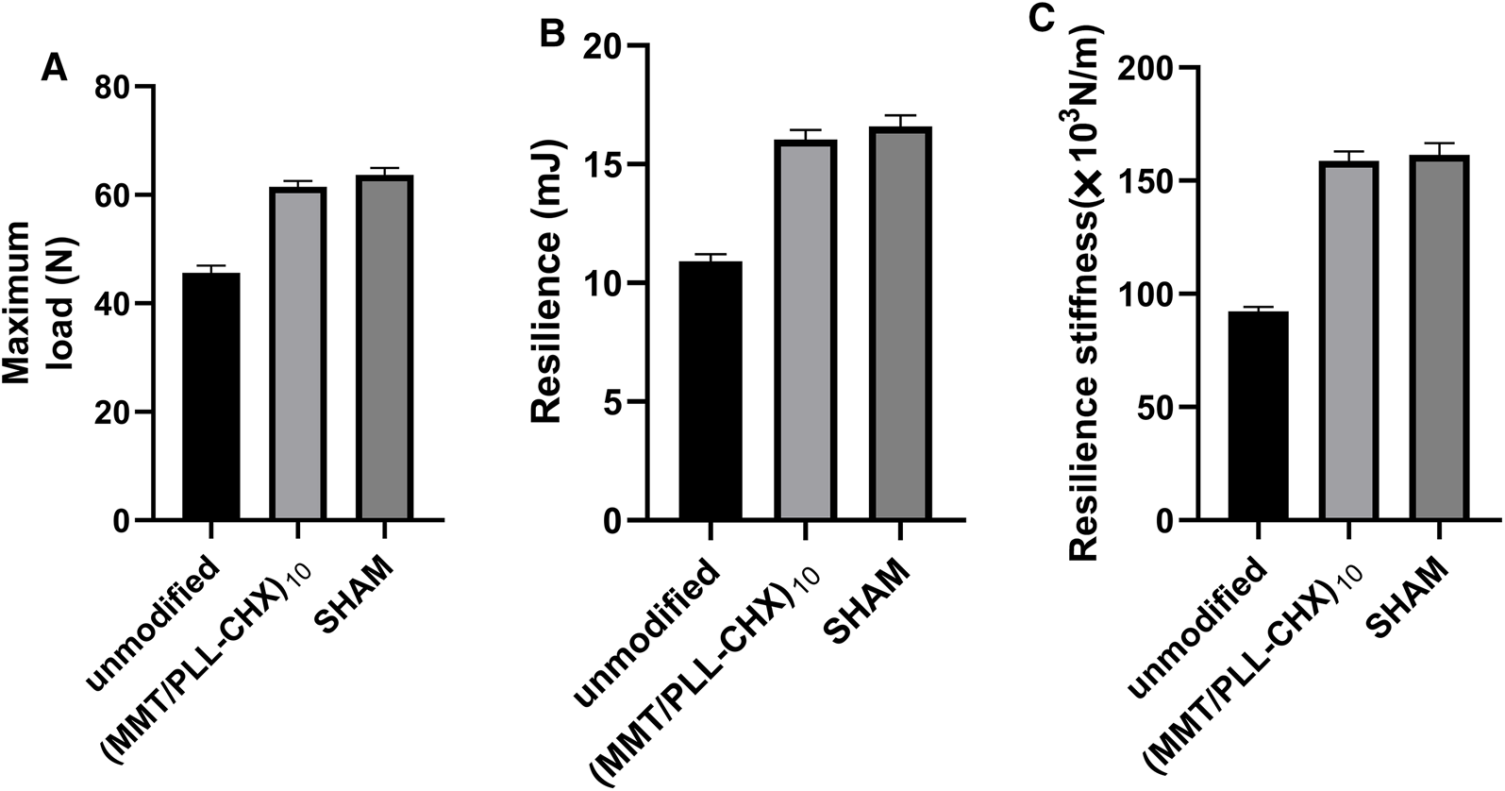
The tibia specimens bending test with different group. **A** The Maximum load. **B** The Resilience. **C** The Resilience stiffness

Infection could affect bone strength, so we used three-point bending experiment to test the integration strength of bone. As demonstrated in the experiments, the Maximum load with unmodified group was 45.63 ± 1.06 N. However, a higher value (61.47 ± 0.92 N) of the (MMT/PLL-CHX)_10_ group was found, which was more than 1.35 times than that of unmodified group (Fig. 10A). The Resilience with unmodified group was 10.90 ± 0.24 mJ, a higher value (16.03 ± 0.33 mJ) of the (MMT/PLL-CHX)_10_ group was found, which was more than 1.47 times than that of unmodified group (Fig. 10B). The Resilience stiffness with unmodified group was 92.23 ± 1.64 × 10^3^ N/m, a higher value (158.80 ± 3.32 µg/mL × 10^3^ N/m) of the (MMT/PLL-CHX)_10_ group was found, which was more than 1.72 times than that of unmodified group (Fig. 10C). However, the above three parameters of the (MMT/PLL-CHX)_10_ group were very close to the SHAM group (P > 0.05).

#### Bone tissue HE staining and Masson trichrome

**Fig. 11:**
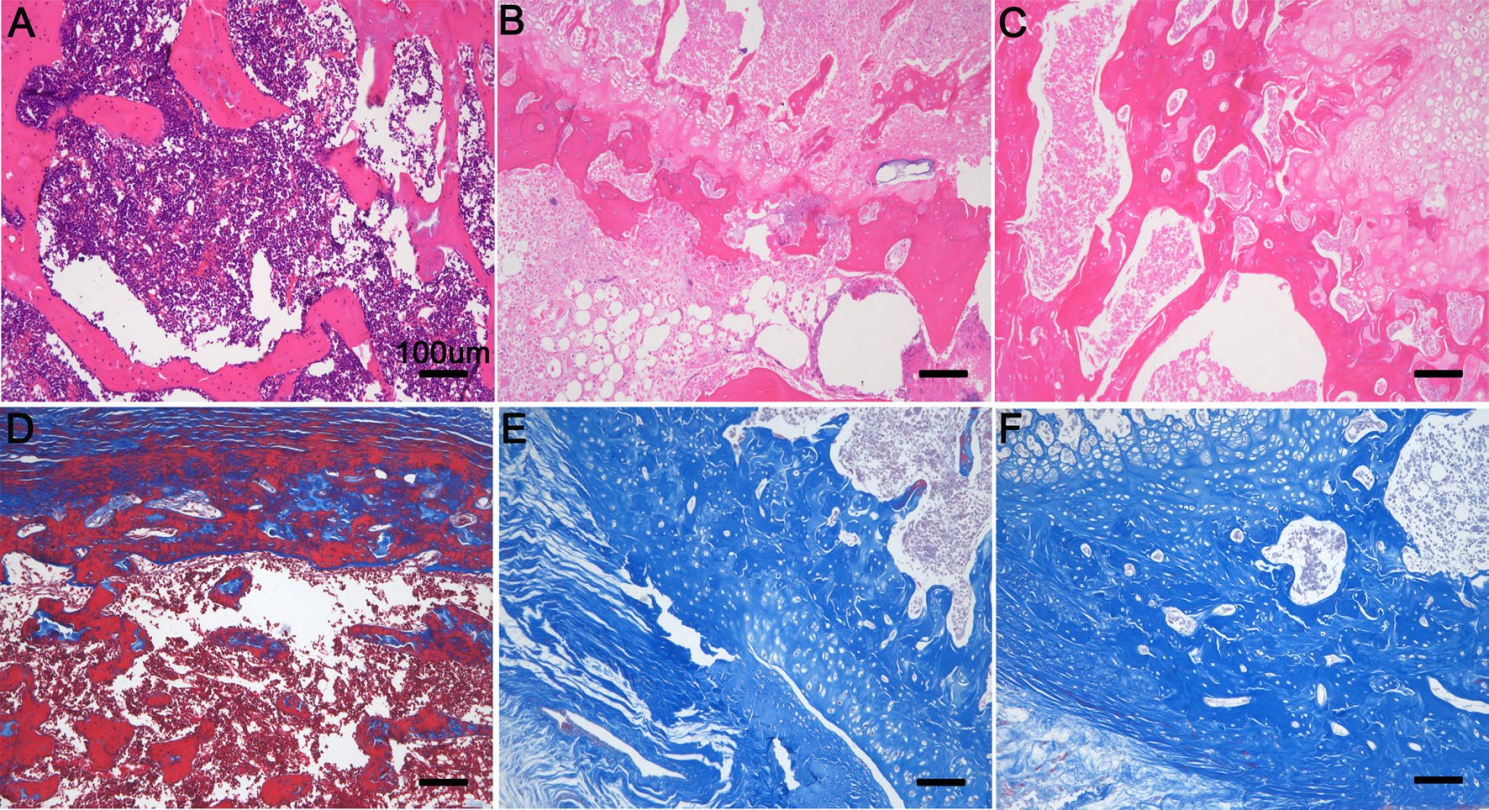
Bone tissue HE staining and Masson trichrome, respectively: **A**, **D** unmodified group, **B**, **E** (MMT/PLL-CHX)_10_ group and **C**, **F** SHAM group

We further did H&E staining and Masson trichrome experiment. We found that there were a large number of inflammatory cells in the bone trabecula of the unmodified group, which confirmed the occurrence of bone infection (Fig. 11A). While the treatment group implanted with (MMT/PLL-CHX)_10_ multilayers coated modified material did not find inflammatory cells and normal bone trabecula was find in here (Fig. 11B). The SHAM group also showed normal bone trabecula (Fig. 11C). A similar phenomenon identified for Masson trichrome. In unmodified group, most areas was stained red due to fibrosis in the bone marrow cavity after infection (Fig. 11D). However, the (MMT/PLL-CHX)_10_ and SHAM group all exhibited normal morphology (Fig. 11E–F).

## Conclusion

All in all, this present research shows that (MMT/PLL-CHX)_10_ multilayer films obtained by layer-by-layer (LbL) assembly exhibited linear growth. Furthermore, CHX depicted on-demand property which was triggered intelligently by CMS or bacterium solution. It is worth noting that the (MMT/PLL-CHX)_10_ multilayer films structure were progressively degraded and showed well concentration-dependent degradation characteristics following incubation with *Staphylococcus aureus* and CMS solution. While the CCK-8 analysis proved that the (MMT/PLL-CHX)_10_ multilayer films possess perfect biocompatibility. We used the shake-flask culture method to test the antibacterial effect of the (MMT/PLL-CHX)_10_ multilayer films. The results showed high levels of bactericidal activity which exhibited a 99% inhibition. It is somewhat encouraging that in the in vivo antibacterial tests, the K-wires coated with (MMT/PLL-CHX)_10_ multilayer films showed lower infections incidence and inflammation than the unmodified group, and all parameters are close to SHAM group. Furthermore, in vivo research demonstrates the potential to provide more robust evidence for the use of this biomaterial to mitigate infections associated with intraosseous implants.

## Data Availability

Not applicable.
